# Relationship between HBV RNA level and pregnancy outcomes among hepatitis B carriers

**DOI:** 10.5937/jomb0-50420

**Published:** 2024-09-06

**Authors:** Manman Zhang, Xin Liao, Heng Wang, Huan Wu, Baofang Zhang

**Affiliations:** 1 Guiyang Public Health Clinical Center, Department of Digestive, Guiyang, China; 2 Guizhou Medical University, Guiyang, China; 3 Guiyang Public Health Clinical Center, Department of Endoscopy, Guiyang, China; 4 Guizhou Medical University, Affiliated Hospital, Department of Infectious Diseases, Guiyang, China

**Keywords:** hepatitis B virus, serum marker, HBV RNA, pregnancy outcomes, intrahepatic cholestasis of pregnancy, virus hepatitisa B, serumski marker, HBV RNA, ishodi trudnoće, intrahepatična holestaza trudnoće

## Abstract

**Background:**

This study aims to investigate the relationship between hepatitis B virus (HBV) RNA level and pregnancy outcomes among hepatitis B carriers.

**Methods:**

This study collected pregnant women who attended the Affiliated Hospital of Guizhou Medical University (Guizhou, China) from June 2020 to June 2023. The levels of HBV DNA, HBV RNA, and HBeAg status in HBV carriers were detected. Pregnancy outcomes including intrahepatic cholestasis of pregnancy (ICP), gestational hypertension (GH), pre-eclampsia, gestational diabetes mellitus (GDM), preterm prelabour rupture of membranes (PPROM), mode of delivery, preterm birth, low birth weight (LBW) and macrosomia.

**Results:**

A total of 562 pregnant women were collected, 203 (36.12%) were infected with HBV. Compared with HBsAg negative, HBsAg positive pregnant women had a higher risk of ICP. There were no significant differences in the rates of GDM, GH, pre-eclampsia, PPROM, preterm birth, LBW, macrosomia, and mode of delivery among women in the two groups. Multivariate logistic regression analysis showed that maternal HBV RNA level (OR = 3.814, 95% CI: 2.036~7.142, P< 0.001) was an independent risk factor for ICP in HBsAg-positive pregnant women. The receiver operating characteristics (ROC) curve revealed that the areas under the curve of HBV RNA for prediction of ICP was 0.8652(95% confidence interval 0.7636-0.9669, P< 0.001).

**Conclusions:**

The HBV RNA level has a significant negative impact on pregnancy outcomes. It may serve as an indicator to guide the prevention of ICP and improve maternal health.

## Abstract (sr)

**Uvod:**

Ova studija ima za cilj da istraži vezu između nivoa RNK virusa hepatitisa B (HBV) i ishoda trudnoće kod nosilaca hepatitisa B.

**Metode:**

Ova studija je prikupila trudnice koje su pohađale pridruženu bolnicu Medicinskog univerziteta Guizhou (Guizhou, Kina) od juna 2020. do juna 2023. Otkriveni su nivoi HBV DNK, HBV RNK i HBeAg statusa kod HBV nosilaca. Ishodi trudnoće uključujući intrahepatičnu holestazu trudnoće (ICP), gestacijsku hipertenziju (GH), preeklampsiju, gestacijski dijabetes melitus (GDM), prevremeno preporođajnu rupturu membrana (PPROM), način porođaja, prevremeni porođaj, nisku porođajnu težinu (LBV) i makrozomiju.

**Rezultati:**

Prikupljeno je ukupno 562 trudnice, 203 (36,12%) su inficirane HBV-om. U poređenju sa HBsAg negativnim, HBsAg pozitivne trudnice su imale veći rizik od ICP. Nije bilo značajnih razlika u stopama GDM, GH, preeklampsije, PPROM, prevremenog porođaja, LBV, makrozomije i načina porođaja među ženama u dve grupe. Multivarijantna logistička regresiona analiza je pokazala da je nivo HBV RNK kod majke (OR = 3,814, 95% CI: 2,036~7,142, P< 0,001) bio nezavisan faktor rizika za ICP kod HBsAg pozitivnih trudnica. Kriva radnih karakteristika prijemnika (ROC) je otkrila da su površine ispod krive HBV RNK za predviđanje ICP-a bile 0,8652 (95% interval poverenja 0,7636-0,9669, P< 0,001).

**Zaključak:**

Nivo HBV RNK ima značajan negativan uticaj na ishod trudnoće. Može poslužiti kao indikator za prevenciju ICP-a i poboljšanje zdravlja majki.

## Introduction

HBV infection is a significant global public health issue, with approximately 296 million people chronically infected with HBV worldwide and more than 800,000 deaths a year from the disease [1]. Traditional HBV serum markers such as HBsAg, HBeAg, and HBV DNA still have limitations in terms of their effectiveness in predicting clinical outcomes, then several new serological markers are being investigated [2]. In recent years, several studies have found that serum HBV RNA can reflect the level and transcriptional activity of HBV covalently closed circular DNA (cccDNA) in the liver, and may be used to monitor the disease progression and predict the prognosis of patients with chronic HBV infection [3].

For a long time, the impact of hepatitis B virus infection on pregnant women’s pregnancy results has been a problem of concern to the majority of patients. The impact of HBV infection on the occurrence of pregnancy complications in pregnant women remains controversial. Some studies suggest that HBV infection is associated with pregnancy complications, while others argue that there is no such relationship.

This study collected blood samples from 203 pregnant women with HBV during pregnancy, HBV DNA, HBV RNA level, and HBeAg status were detected. GH, pre-eclampsia, GDM, delivery mode, preterm birth, LBW, and macrosomia were recorded in hepatitis B carriers. To investigate the relationship between HBV RNA, a novel clinical marker, and pregnancy complications.

## Materials and methods

### Study design and populations

This study collected pregnant women admitted to the Affiliated Hospital of Guizhou Medical University from June 2020 to June 2023. The diagnosis of hepatitis B carriers was consistent with the 2022 update of the Guideline of Prevention and Treatment for Chronic Hepatitis B in China [4].

Inclusion Criteria: 1) patients >18 years; 2) patients able to attend follow-up visits as required and signed an informed consent form.

Exclusion Criteria: 1) patients with HIV infection and hepatitis C virus infection and other infectious diseases; 2) patients with non-alcoholic steatohepatitis, autoimmune hepatitis, and cirrhosis and other hepatobiliary diseases; 3) patients with malignant tumors and hematological diseases; 4) patients who have experienced miscarriage, termination of pregnancy, stillbirth, or multiple pregnancies.

Informed consent was obtained from all subjects and this study protocol was approved by the Ethics Committee of the Affiliated Hospital of Guizhou Medical University (Guizhou, China) and performed in accordance with the relevant provisions of the Helsinki Declaration.

### Data collection

The general data and clinical information, including Age, ethnicity, parity status, HBeAg status, HBV DNA, and HBV RNA were collected. All blood samples were collected during pregnancy. The OMEGA Viral RNA Kit (Omega Bio-Tek, Biotek Winooski, VT, USA) was used for the extraction of HBV RNA from serum. Serum RNA was measured by quantitative reverse transcription-polymerase chain reaction (RT-qPCR). The specific primers (including HBV RNA RT primer 5-ACC ACG CTA TCG CTA CTC AC (t17) GWA GCT C) used were designed according to van Bömmel et al. [5] HBV DNA was measured using a 7,500 Real-Time PCR System (Applied Biosystems, Foster City, CA, USA). The HBeAg status was examined with HBeAg ELISA Kit (Jingmei Biotechnology, Jiangsu, China) using the Synergy™ H4 Multi-function microplate reader (BioTek, Biotek Winooski, VT, USA). HBV DNA <100 IU/mL, HBeAg <0.5 PEIU/mL was reported as negative.

### Statistical analysis

Statistic Package for Social Science (SPSS) version 27.0 (SPSS Inc., Chicago, IL, USA) was used to perform statistical analyses. Rates (n, %) indicate categorical variables and were analyzed by chi-square test. Logistic regression analysis was used to determine risk factors of ICP. The ROC curve was constructed to determine the clinical diagnostic value of ICP. Serum HBV RNA levels and HBV DNA levels were log-Transformed. A two-sided *P*<0.05 was considered a statistically significant difference.

## Results

### Patient population

A total of 562 pregnant women were collected, and 203 (36.12%) were infected with HBV. The basic characteristics of patients are shown in Table 1.

**Table 1:** Basic maternal demographics.

Parameters	Whole cohort<br>N=562	HBsAg positive<br>N=203	HBsAg negative<br>N=359	*P*
Age, years				<0.001
<35, n (%)	429	181 (32.21)	248 (44.13)	
≥35, n (%)	133	22 (3.91)	111 (19.75)	
Ethnic groups, n (%)				0.116
Han	445	168 (29.89)	277 (49.29)	
Others	117	35 (6.23)	82(14.59)	
Parity status, n (%)				<0.001
1	382	120 (21.35)	262 (46.62)	
2	180	83 (14.77)	97 (17.26)	
in Vitro Fertilisation				0.845
yes	116	41 (7.30)	75 (13.34)	
no	446	162 (28.83)	284 (50.53)	

### Comparison of pregnancy outcomes between HBsAg positive and negative pregnant women

The rates of cholestasis during pregnancy in HBsAg-positive pregnant women were significantlyhigher than those in HBsAg-negative pregnant women (χ^2^=10.200, P=0.001, Table 2). There was no significant difference in other pregnancy outcomes between the two groups (P > 0.05, Table 2).

**Table 2:** Comparison of the pregnancy outcomes HBsAg positive and non-HBV controls. Pregnancy outcomes including intrahepatic cholestasis of pregnancy (ICP), gestational hypertension (GH), pre-eclampsia, gestational diabetes mellitus (GDM), preterm prelabour rupture of membranes (PPROM)

Parameters	HBsAg positive<br>N=203	HBsAg negative<br>N=359	χ^2^	P
ICP, n (%)	15	7	10.200	0.001
GDM, n (%)	32	75	2.212	0.137
GH, n (%)	5	3	2.447	0.145
Pre-eclampsia, n (%)	8	15	0.019	0.891
PPROM, n (%)	27	42	0.309	0.578
Preterm birth, n (%)	18	51	3.432	0.064
Vaginal delivery, n (%)	155	292	1.978	0.160
Birth weight, (grams), n (%)				
≤2500 g, n (%)	8	29	3.642	0.056
≥4000 g, n (%)	4	8	0.076	0.782

### Factors related to ICP

Table 3 shows that by univariate logistic regression analysis, risk factors affecting ICP include: HBV RNA level (OR =3.449, 95% CI: 2.005~5.932, P< 0.001) and HBV DNA level (OR =2.066, 95% CI: 1.338~3.188, P< 0.001). Similarly, HBV RNA level (OR = 3.814, 95% CI: 2.036~7.142, P< 0.001) and HBV DNA level (OR = 2.392, 95% CI: 1.345~4.256, P=0.003) were independently risk factors for ICP by multivariate logistic regression analysis (Table 3). HBsAg-positive pregnant women whose HBV DNA and HBV RNA were higher than 6 log10 IU/mL were found to be at risk of ICP. The results are summarized in Table 4.

**Table 3:** Logistic regression analysis.

	Univariable			Multivariable		
	OR	CI-95%CI	*p*	OR	CI-95%CI	*p*
Age	1.013	(0.892~1.150)	0.846			
Parity status	1.261	(0.439~3.623)	0.666			
Ethnic groups	1.757	(0.381~8.106)	0.470			
HBV RNA	3.449	(2.005~5.932)	<0.001	3.814	(2.036~7.142)	0.001
HBV DNA	2.066	(1.338~3.188)	<0.001	2.392	(1.345~4.256)	0.003
HBeAg						
HBeAg pos	1.107	(0.386~3.177)	0.850			
HBeAg neg	reference					

**Table 4:** Pregnancy outcomes with different maternal HBV RNA levels.

	n	ICP, n (%)	p
HBV RNA (log10 IU/mL)			<0.001
≤3	12	0 (0%)	
3~6	138	4 (2.90%)	
≥6	53	11 (20.75%)	
HBV DNA (log10 IU/mL)			0.004
≤3	22	0 (0%)	
3~6	125	5 (4%)	
≥6	56	10 (17.86%)	

### Diagnosis value of HBV RNA in ICP

**Figure 1:**
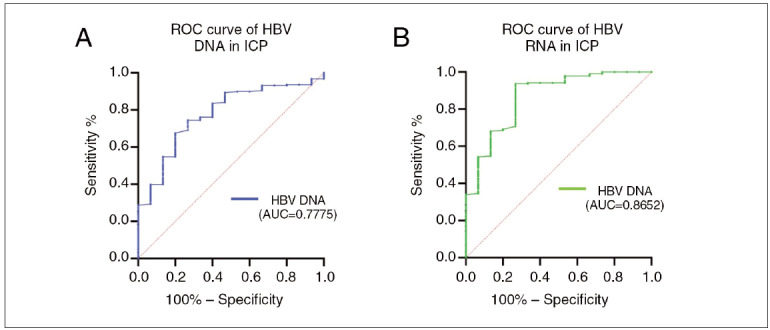
Receiver operating characteristics (ROC) curves of HBV RNA and HBV DNA discriminating HBsAg positive from ICP patients. (A) HBV DNA level manifested an area under the curve (AUC) value of 0.7775 which was lower than 0.8 and P < 0.05. (B) HBV RNA level manifested an area under the curve (AUC) value of 0.8652 which was greater than 0.8 and P < 0.05, suggesting HBV RNA maybe a potential diagnostic candidate for ICP. ICP, Pregnancy outcomes including intrahepatic cholestasis of pregnancy.

Figure 1 shows the performance of the HBV RNA and HBV DNA in predicting ICP. For the prediction of HBV infection, the areas under the curves (AUCs) of HBV RNA and HBV DNA for prediction of ICP were 0.8652 (95% CI = 0.7636~0.9669, P < 0.001) and 0.7775 (95% CI = 0.6672~0.8877, P < 0.001), respectively.

## Discussion

HBV cccDNA is a unique intermediate that forms during HBV replication and serves as a transcriptional template for various mRNAs of different sizes, including pre-genomic RNA and pre-core RNA. Understanding HBV cccDNA is crucial for a comprehensive understanding of HBV replication and infection status [6] [7]. Increasingly, studies have shown that HBV RNA can be used as an alternative marker for HBV cccDNA to guide CHB treatment and assess disease progression. However, its role has not been systematically studied in pregnant women with HBV infection [8] [9].

Wan et al. [10] found that gestational hypertension (GH), cesarean section, macrosomia, and preterm birth risk were associated with maternal HBsAg. Cai et al. reported that the increased risk of premature rupture of membranes (PROM) and intrahepatic cholestasis of pregnancy (ICP) are related to hepatitis B carriers during pregnancy [11]. However, study did not find a relationship between HBV infection and adverse pregnancy outcomes [12] [13]. Currently, studies on pregnancy outcomes in women with hepatitis B infection primarily focus on analyzing HBsAg, HBV DNA, and HBeAg status. Few studies have used HBV RNA as a parameter to evaluate pregnancy outcomes. As a new virological marker, HBV RNA can reflect the replication of the virus, and its detection may guide managing pregnant women infected with hepatitis B. In this study, we compared the pregnancy outcomes of 203 HBsAg-positive and 359 HBsAg-negative pregnant women, examining outcomes such as ICP, GH, preeclampsia, gestational diabetes mellitus (GDM), PROM, mode of delivery, preterm birth, low birth weight (LBW), and macrosomia. The results indicate that HBV RNA has predictive value for ICP in pregnant women infected with hepatitis B.

ICP is an idiopathic condition in pregnancy characterized by pruritus and/or abnormal liver function in pregnant women, along with elevated total bile acid levels and the absence of other liver and bile diseases. This condition primarily threatens newborn outcomes, including amniotic fluid contamination, fetal distress, preterm delivery, and even sudden fetal death in utero [14]. The concentration of bile acid is used to diagnose and monitor the general condition of pregnant women with intrahepatic cholestasis. The etiology of ICP is not clear but is likely due to a combination of genetic predisposition factors (such as hepatobiliary transporter variants), hormonal factors, and environmental factors [15] [16] [17] [18]. Some studies have identified sodium-taurocholate cotransporting polypeptide (NTCP) as a specific receptor for HBV-infected hepatocytes, and changes in hormone levels during pregnancy may increase the risk of ICP in women infected with HBV [19]. Our study found that HBV RNA is a significant risk factor for ICP (OR = 3.814, 95% CI: 2.036–7.142, P < 0.001). The area under the curve (AUC) for HBV RNA was 0.8652 (95% CI: 0.7636–0.9669, P < 0.001), indicating its potential to predict the occurrence of ICP in HBV-infected pregnant women.

However, several limitations are present in this study. Firstly, the sample size was small, underscoring the need for larger studies to validate our findings. Secondly, the study population consisted entirely of Chinese individuals, raising questions about the generalizability of our results to other countries and ethnicities. Thirdly, quantitative methods for HBV RNA are still evolving, which could potentially influence our experimental results. Therefore, the development of more standardized detection methods and reagents is urgently warranted to enhance the accuracy and reliability of future studies.

## Conclusion

This study revealed the HBV RNA level has a significant negative impact on pregnancy outcomes. Based on the results of the study, we can conclude that high levels of HBV RNA during pregnancy may be a risk factor for the ICP and that may predict the ICP.

## Dodatak

### Ethics statement

This study protocol was approved by the Ethics Committee of the Affiliated Hospital of Guizhou Medical University (Guizhou, China) and performed in accordance with the relevant provisions of the Helsinki Declaration.

### Funding

This work was supported by the National Natural Science Foundation of China (8206030679) and 2023 Guizhou Basic Research Plan (Natural Science) Project (Qianhe Foundation -zk[2023] General 229).

### Conflict of interest statement

All the authors declare that they have no conflict of interest in this work.
